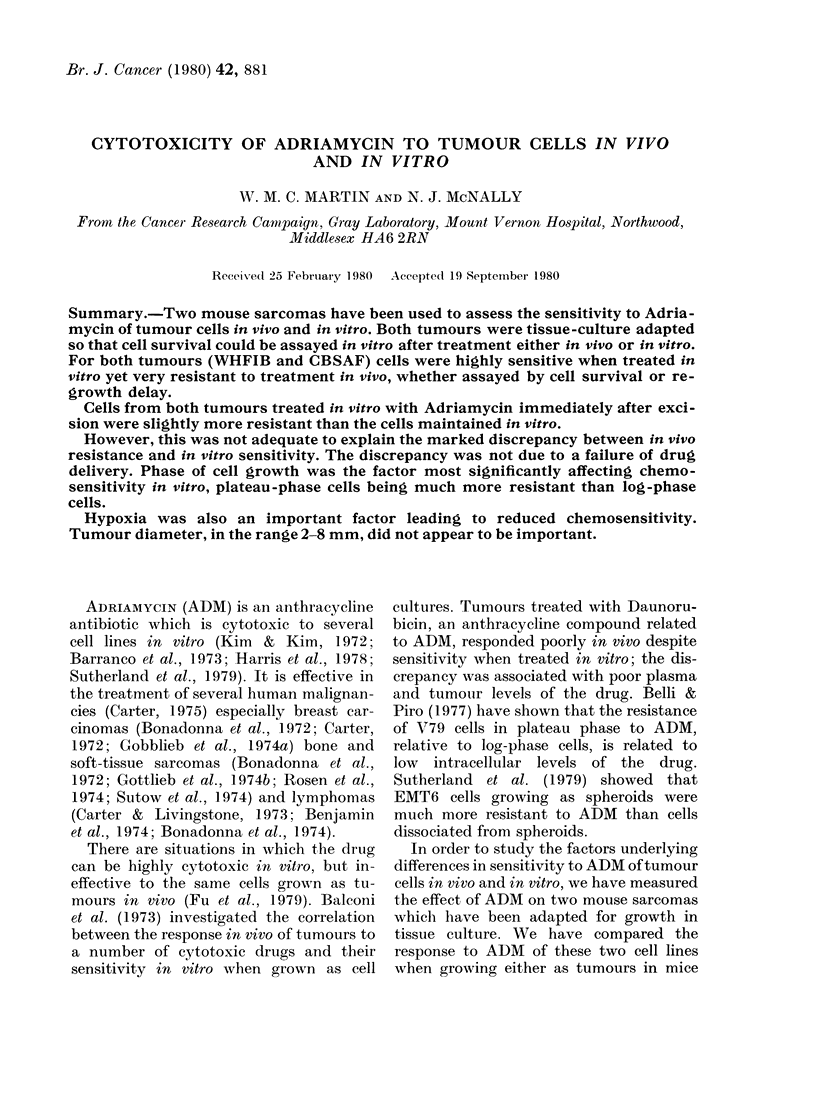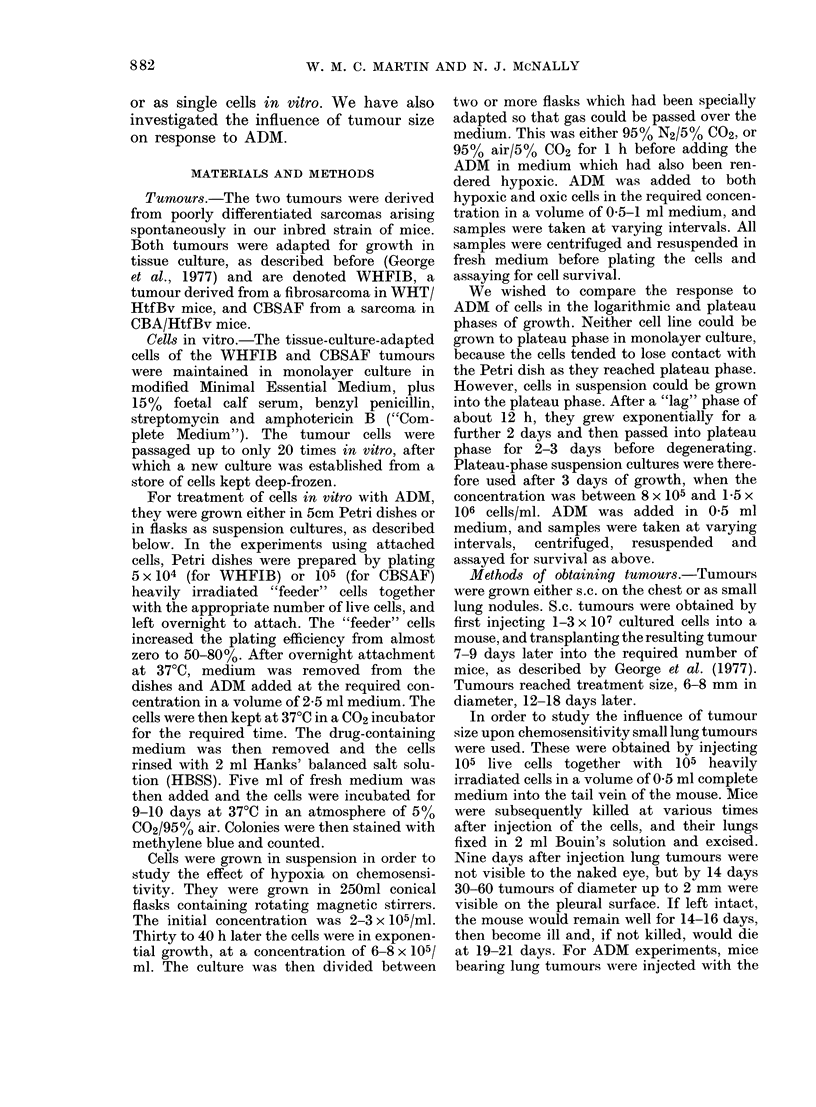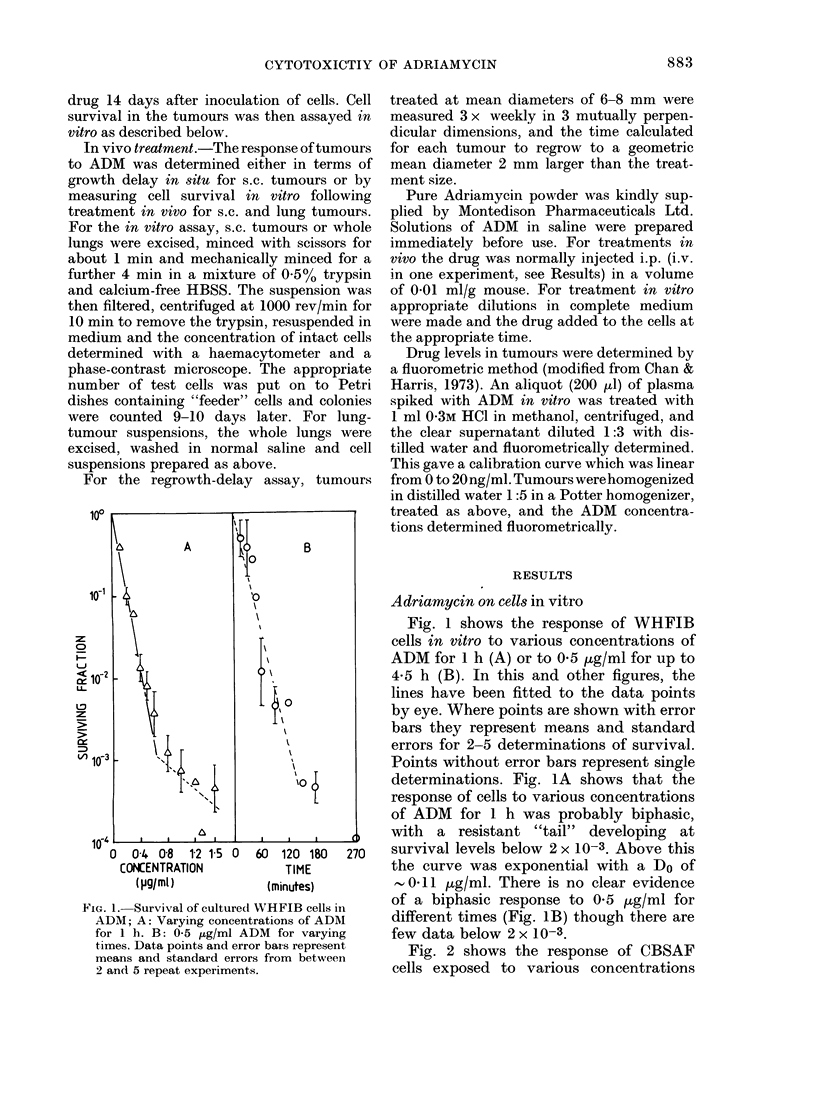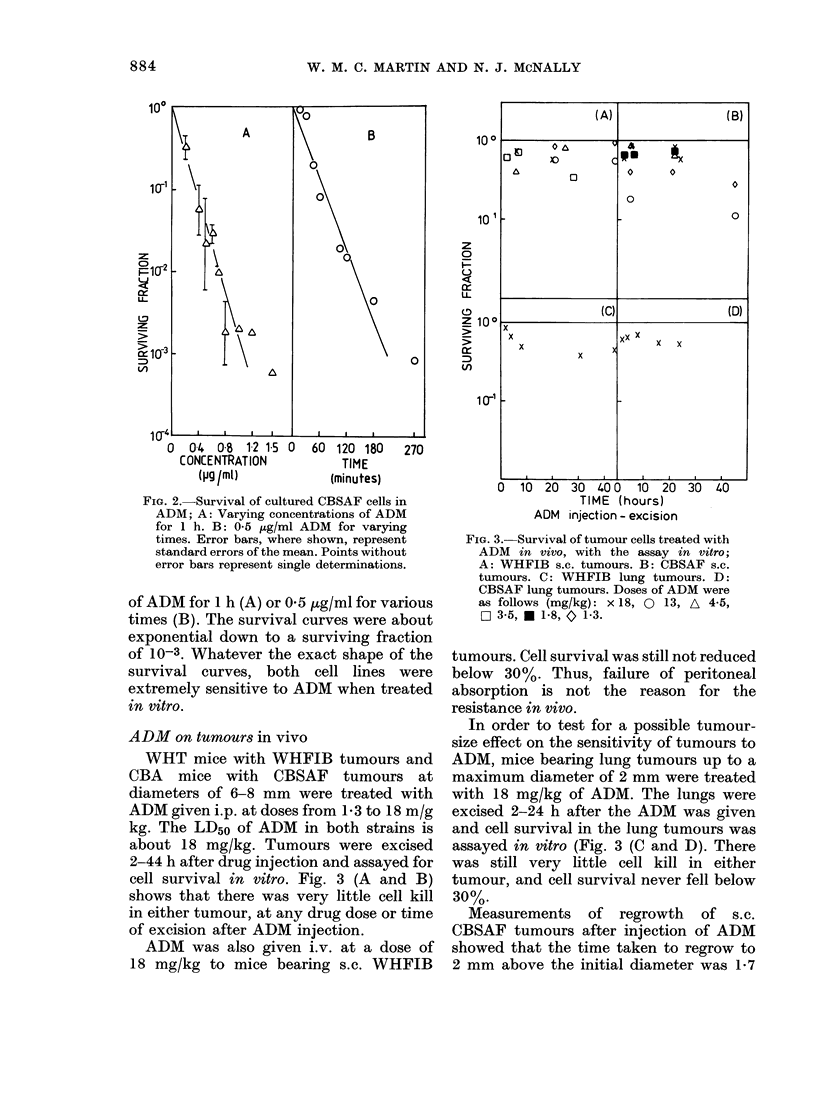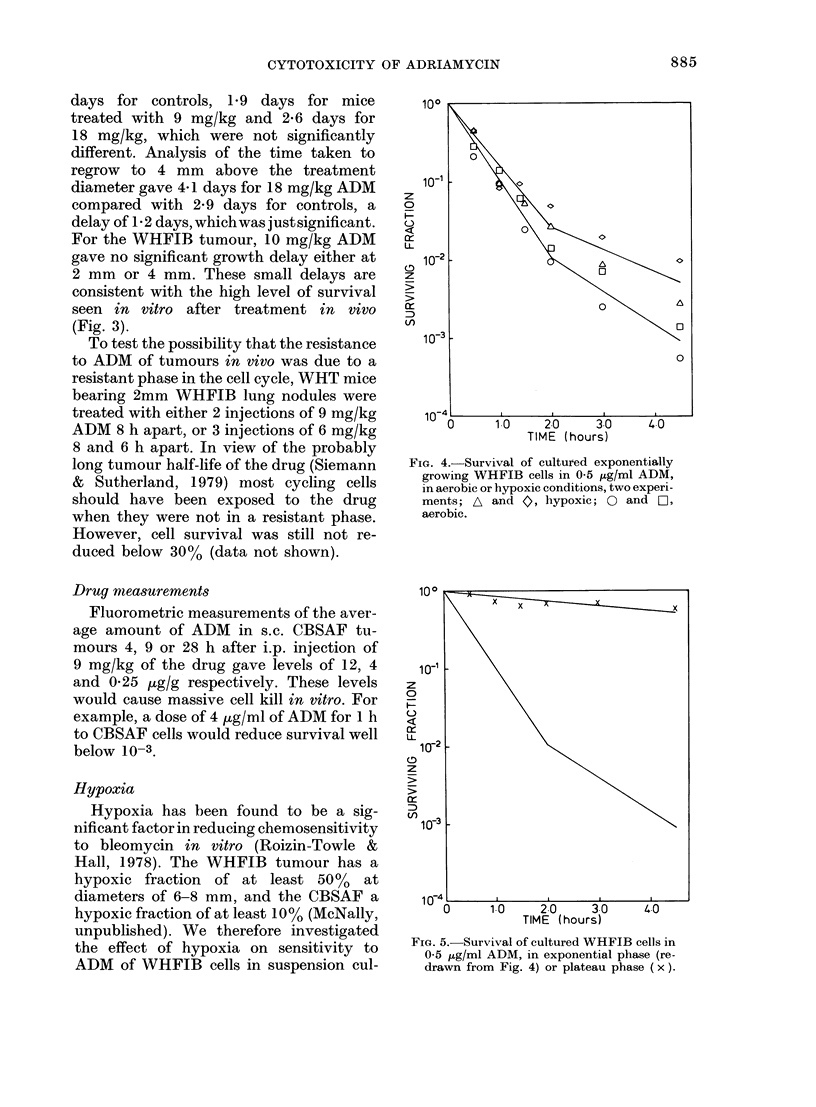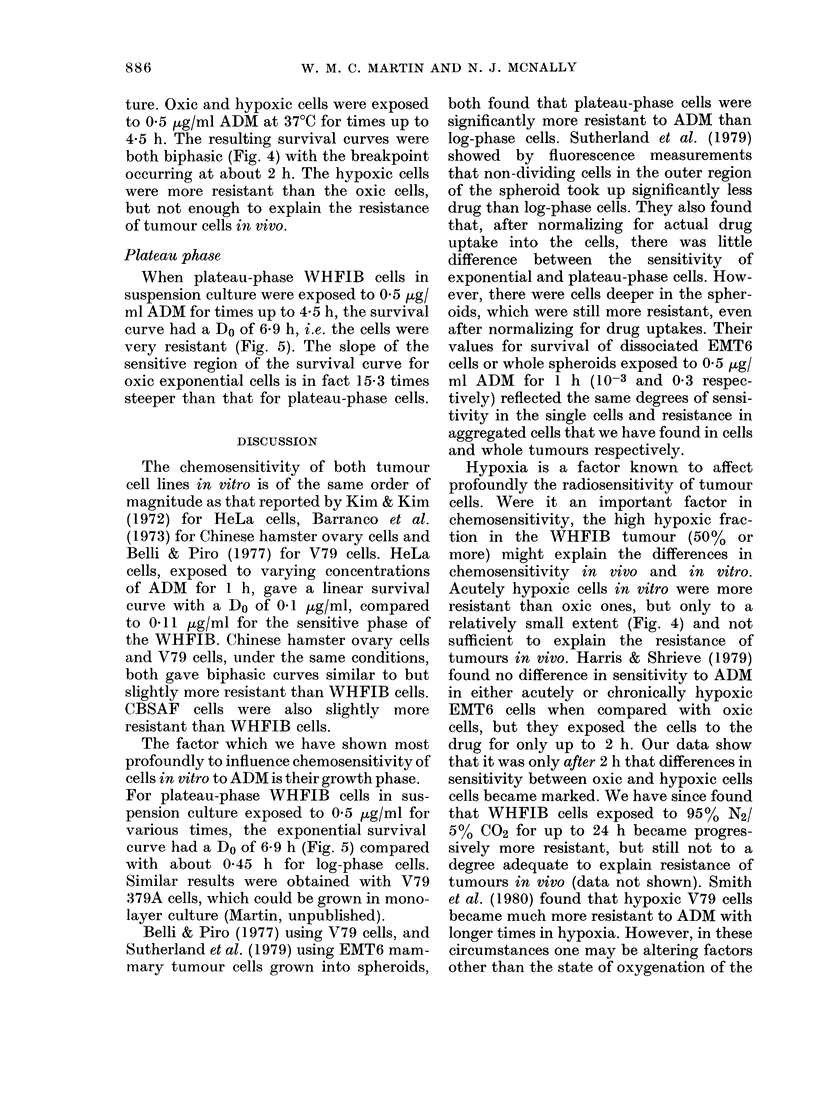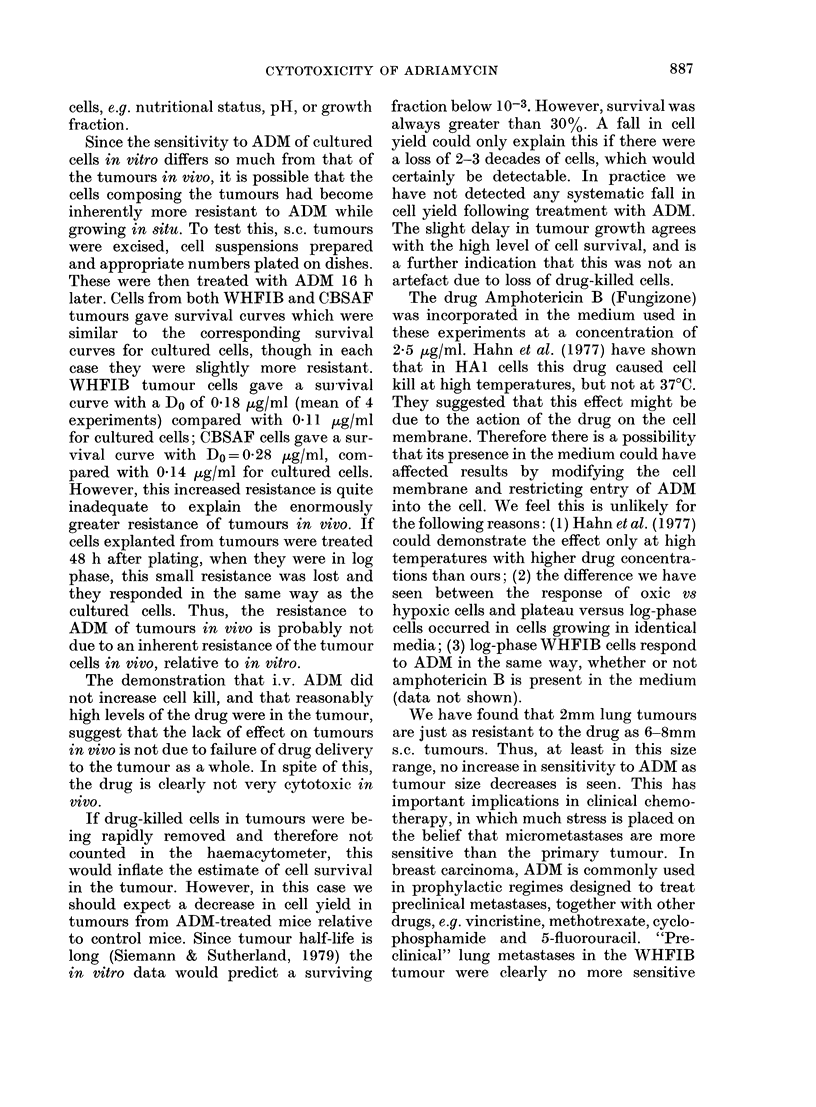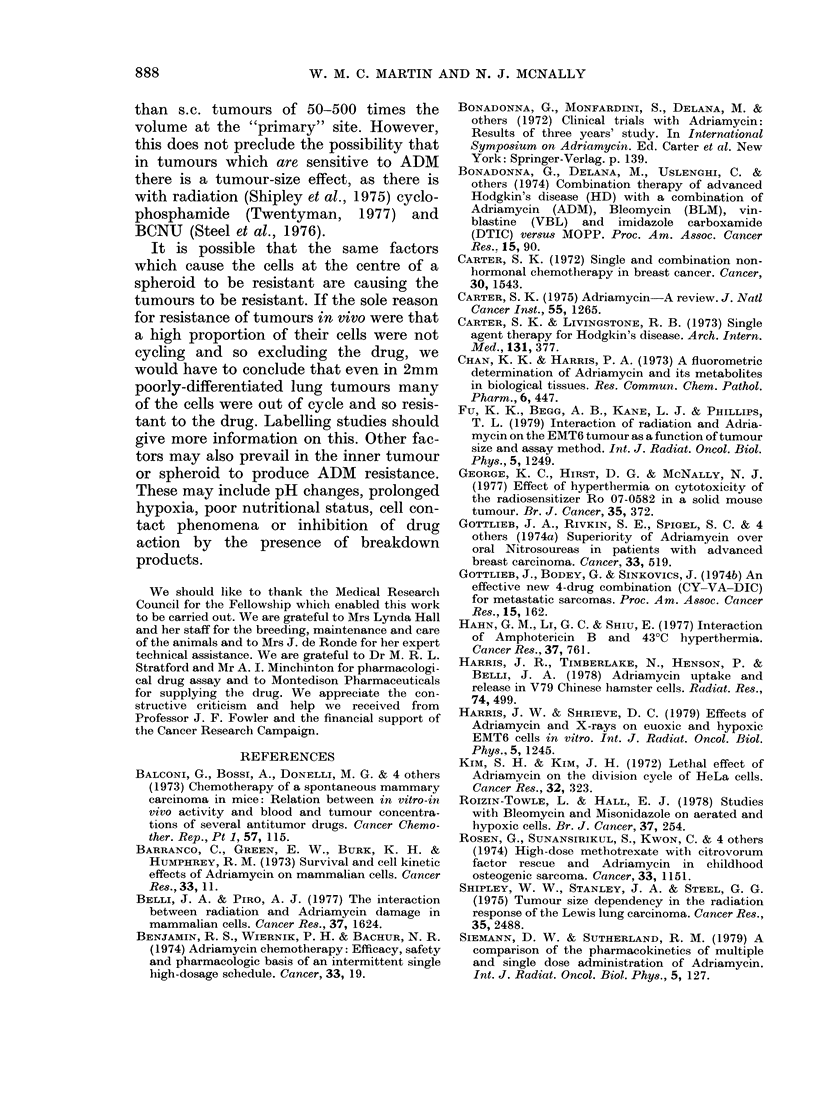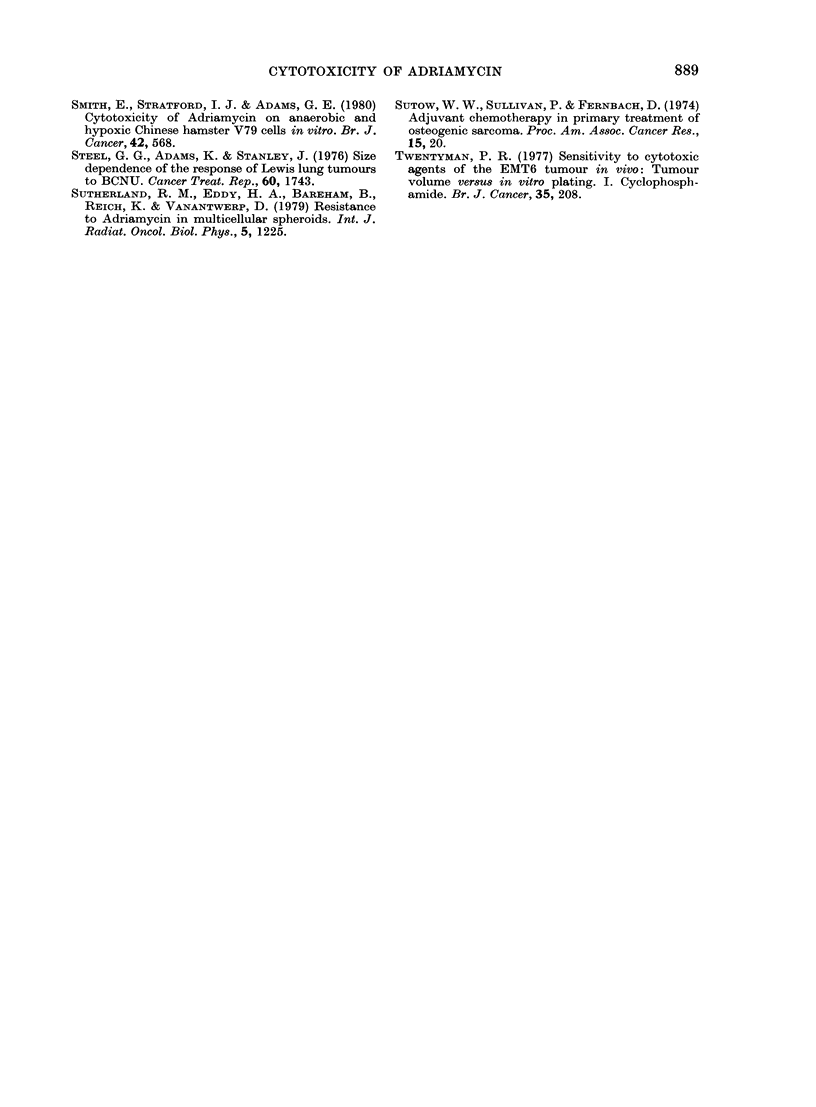# Cytotoxicity of adriamycin to tumour cells in vivo and in vitro.

**DOI:** 10.1038/bjc.1980.336

**Published:** 1980-12

**Authors:** W. M. Martin, N. J. McNally

## Abstract

Two mouse sarcomas have been used to assess the sensitivity to Adriamycin of tumour cells in vivo and in vitro. Both tumours were tissue-culture adapted so that cell survival could be assayed in vitro after treatment either in vivo or in vitro. For both tumours (WHFIB and CBSAF) cells were highly sensitive when treated in vitro yet very resistant to treatment in vivo, whether assayed by cell survival or regrowth delay. Cells from both tumours treated in vitro with Adriamycin immediately after excision were slightly more resistant than the cells maintained in vitro. However, this was not adequate to explain the marked discrepancy between in vivo resistance and in vitro sensitivity. The discrepancy was not due to a failure of drug delivery. Phase of cell growth was the factor was most significantly affecting chemosensitivity in vitro, plateau-phase cells being much more resistant than log-phase cells. Hypoxia was also an important factor leading to reduced chemosensitivity. Tumour diameter, in the range 2-8 mm, did not appear to be important.


*Br. J. Cancer* (1980) **42**, 881

### CYTOTOXICITY OF ADRIAMYCIN TO TUMOUR CELLS *IN VIVO* AND *IN VITRO*

W. M. C. MARTIN AND N. J. McNALLY

*From the Cancer Research Campaign, Gray Laboratory, Mount Vernon Hospital, Northwood, Middlesex HA6 2RN*

Received 25 February 1980 Accepted 19 September 1980

**Summary.—Two mouse sarcomas have been used to assess the sensitivity to Adriamycin of tumour cells *in vivo* and *in vitro*. Both tumours were tissue-culture adapted so that cell survival could be assayed *in vitro* after treatment either *in vivo* or *in vitro*. For both tumours (WHFIB and CBSAF) cells were highly sensitive when treated *in vitro* yet very resistant to treatment *in vivo*, whether assayed by cell survival or regrowth delay.**

**Cells from both tumours treated *in vitro* with Adriamycin immediately after excision were slightly more resistant than the cells maintained *in vitro*.**

**However, this was not adequate to explain the marked discrepancy between *in vivo* resistance and *in vitro* sensitivity. The discrepancy was not due to a failure of drug delivery. Phase of cell growth was the factor most significantly affecting chemosensitivity *in vitro*, plateau-phase cells being much more resistant than log-phase cells.**

**Hypoxia was also an important factor leading to reduced chemosensitivity. Tumour diameter, in the range 2–8 mm, did not appear to be important.**

ADRIAMYCIN (ADM) is an anthracycline antibiotic which is cytotoxic to several cell lines *in vitro* (Kim & Kim, 1972; Barranco *et al.*, 1973; Harris *et al.*, 1978; Sutherland *et al.*, 1979). It is effective in the treatment of several human malignancies (Carter, 1975) especially breast carcinomas (Bonadonna *et al.*, 1972; Carter, 1972; Gobblieb *et al.*, 1974*a*) bone and soft-tissue sarcomas (Bonadonna *et al.*, 1972; Gottlieb *et al.*, 1974*b*; Rosen *et al.*, 1974; Sutow *et al.*, 1974) and lymphomas (Carter & Livingstone, 1973; Benjamin *et al.*, 1974; Bonadonna *et al.*, 1974).

There are situations in which the drug can be highly cytotoxic *in vitro*, but ineffective to the same cells grown as tumours *in vivo* (Fu *et al.*, 1979). Balconi *et al.* (1973) investigated the correlation between the response *in vivo* of tumours to a number of cytotoxic drugs and their sensitivity *in vitro* when grown as cell cultures. Tumours treated with Daunorubicin, an anthracycline compound related to ADM, responded poorly *in vivo* despite sensitivity when treated *in vitro*; the discrepancy was associated with poor plasma and tumour levels of the drug. Belli & Piro (1977) have shown that the resistance of V79 cells in plateau phase to ADM, relative to log-phase cells, is related to low intracellular levels of the drug. Sutherland *et al.* (1979) showed that EMT6 cells growing as spheroids were much more resistant to ADM than cells dissociated from spheroids.

In order to study the factors underlying differences in sensitivity to ADM of tumour cells *in vivo* and *in vitro*, we have measured the effect of ADM on two mouse sarcomas which have been adapted for growth in tissue culture. We have compared the response to ADM of these two cell lines when growing either as tumours in mice or as single cells *in vitro*. We have also investigated the influence of tumour size on response to ADM.

### MATERIALS AND METHODS

*Tumours.*—The two tumours were derived from poorly differentiated sarcomas arising spontaneously in our inbred strain of mice. Both tumours were adapted for growth in tissue culture, as described before (George *et al.*, 1977) and are denoted WHFIB, a tumour derived from a fibrosarcoma in WHT/HtfBv mice, and CBSAF from a sarcoma in CBA/HtfBv mice.

*Cells* in vitro.—The tissue-culture-adapted cells of the WHFIB and CBSAF tumours were maintained in monolayer culture in modified Minimal Essential Medium, plus 15% foetal calf serum, benzyl penicillin, streptomycin and amphotericin B ("Complete Medium"). The tumour cells were passaged up to only 20 times *in vitro*, after which a new culture was established from a store of cells kept deep-frozen.

For treatment of cells *in vitro* with ADM, they were grown either in 5cm Petri dishes or in flasks as suspension cultures, as described below. In the experiments using attached cells, Petri dishes were prepared by plating $5 \times 10^4$ (for WHFIB) or $10^5$ (for CBSAF) heavily irradiated "feeder" cells together with the appropriate number of live cells, and left overnight to attach. The "feeder" cells increased the plating efficiency from almost zero to 50–80%. After overnight attachment at 37°C, medium was removed from the dishes and ADM added at the required concentration in a volume of 2·5 ml medium. The cells were then kept at 37°C in a $CO_2$ incubator for the required time. The drug-containing medium was then removed and the cells rinsed with 2 ml Hanks' balanced salt solution (HBSS). Five ml of fresh medium was then added and the cells were incubated for 9–10 days at 37°C in an atmosphere of 5% $CO_2$/95% air. Colonies were then stained with methylene blue and counted.

Cells were grown in suspension in order to study the effect of hypoxia on chemosensitivity. They were grown in 250ml conical flasks containing rotating magnetic stirrers. The initial concentration was $2\text{–}3 \times 10^5$/ml. Thirty to 40 h later the cells were in exponential growth, at a concentration of $6\text{–}8 \times 10^5$/ml. The culture was then divided between two or more flasks which had been specially adapted so that gas could be passed over the medium. This was either 95% $N_2$/5% $CO_2$, or 95% air/5% $CO_2$ for 1 h before adding the ADM in medium which had also been rendered hypoxic. ADM was added to both hypoxic and oxic cells in the required concentration in a volume of 0·5–1 ml medium, and samples were taken at varying intervals. All samples were centrifuged and resuspended in fresh medium before plating the cells and assaying for cell survival.

We wished to compare the response to ADM of cells in the logarithmic and plateau phases of growth. Neither cell line could be grown to plateau phase in monolayer culture, because the cells tended to lose contact with the Petri dish as they reached plateau phase. However, cells in suspension could be grown into the plateau phase. After a "lag" phase of about 12 h, they grew exponentially for a further 2 days and then passed into plateau phase for 2–3 days before degenerating. Plateau-phase suspension cultures were therefore used after 3 days of growth, when the concentration was between $8 \times 10^5$ and $1{\cdot}5 \times 10^6$ cells/ml. ADM was added in 0·5 ml medium, and samples were taken at varying intervals, centrifuged, resuspended and assayed for survival as above.

*Methods of obtaining tumours.*—Tumours were grown either s.c. on the chest or as small lung nodules. S.c. tumours were obtained by first injecting $1\text{–}3 \times 10^7$ cultured cells into a mouse, and transplanting the resulting tumour 7–9 days later into the required number of mice, as described by George *et al.* (1977). Tumours reached treatment size, 6–8 mm in diameter, 12–18 days later.

In order to study the influence of tumour size upon chemosensitivity small lung tumours were used. These were obtained by injecting $10^5$ live cells together with $10^5$ heavily irradiated cells in a volume of 0·5 ml complete medium into the tail vein of the mouse. Mice were subsequently killed at various times after injection of the cells, and their lungs fixed in 2 ml Bouin's solution and excised. Nine days after injection lung tumours were not visible to the naked eye, but by 14 days 30–60 tumours of diameter up to 2 mm were visible on the pleural surface. If left intact, the mouse would remain well for 14–16 days, then become ill and, if not killed, would die at 19–21 days. For ADM experiments, mice bearing lung tumours were injected with the drug 14 days after inoculation of cells. Cell survival in the tumours was then assayed *in vitro* as described below.

In vivo *treatment*.—The response of tumours to ADM was determined either in terms of growth delay *in situ* for s.c. tumours or by measuring cell survival *in vitro* following treatment *in vivo* for s.c. and lung tumours. For the *in vitro* assay, s.c. tumours or whole lungs were excised, minced with scissors for about 1 min and mechanically minced for a further 4 min in a mixture of 0·5% trypsin and calcium-free HBSS. The suspension was then filtered, centrifuged at 1000 rev/min for 10 min to remove the trypsin, resuspended in medium and the concentration of intact cells determined with a haemacytometer and a phase-contrast microscope. The appropriate number of test cells was put on to Petri dishes containing "feeder" cells and colonies were counted 9–10 days later. For lung-tumour suspensions, the whole lungs were excised, washed in normal saline and cell suspensions prepared as above.

For the regrowth-delay assay, tumours treated at mean diameters of 6–8 mm were measured 3 × weekly in 3 mutually perpendicular dimensions, and the time calculated for each tumour to regrow to a geometric mean diameter 2 mm larger than the treatment size.

FIG. 1.—Survival of cultured WHFIB cells in ADM; A: Varying concentrations of ADM for 1 h. B: 0·5 μg/ml ADM for varying times. Data points and error bars represent means and standard errors from between 2 and 5 repeat experiments.

Pure Adriamycin powder was kindly supplied by Montedison Pharmaceuticals Ltd. Solutions of ADM in saline were prepared immediately before use. For treatments *in vivo* the drug was normally injected i.p. (i.v. in one experiment, see Results) in a volume of 0·01 ml/g mouse. For treatment *in vitro* appropriate dilutions in complete medium were made and the drug added to the cells at the appropriate time.

Drug levels in tumours were determined by a fluorometric method (modified from Chan & Harris, 1973). An aliquot (200 μl) of plasma spiked with ADM *in vitro* was treated with 1 ml 0·3M HCl in methanol, centrifuged, and the clear supernatant diluted 1:3 with distilled water and fluorometrically determined. This gave a calibration curve which was linear from 0 to 20 ng/ml. Tumours were homogenized in distilled water 1:5 in a Potter homogenizer, treated as above, and the ADM concentrations determined fluorometrically.

### RESULTS

### *Adriamycin on cells* in vitro

Fig. 1 shows the response of WHFIB cells *in vitro* to various concentrations of ADM for 1 h (A) or to 0·5 μg/ml for up to 4·5 h (B). In this and other figures, the lines have been fitted to the data points by eye. Where points are shown with error bars they represent means and standard errors for 2–5 determinations of survival. Points without error bars represent single determinations. Fig. 1A shows that the response of cells to various concentrations of ADM for 1 h was probably biphasic, with a resistant "tail" developing at survival levels below $2 \times 10^{-3}$. Above this the curve was exponential with a $D_0$ of ~0·11 μg/ml. There is no clear evidence of a biphasic response to 0·5 μg/ml for different times (Fig. 1B) though there are few data below $2 \times 10^{-3}$.

Fig. 2 shows the response of CBSAF cells exposed to various concentrations

FIG. 2.—Survival of cultured CBSAF cells in ADM; A: Varying concentrations of ADM for 1 h. B: 0·5 μg/ml ADM for varying times. Error bars, where shown, represent standard errors of the mean. Points without error bars represent single determinations.

FIG. 3.—Survival of tumour cells treated with ADM *in vivo*, with the assay *in vitro*; A: WHFIB s.c. tumours. B: CBSAF s.c. tumours. C: WHFIB lung tumours. D: CBSAF lung tumours. Doses of ADM were as follows (mg/kg): × 18, ○ 13, △ 4·5, □ 3·5, ■ 1·8, ◇ 1·3.

of ADM for 1 h (A) or 0·5 μg/ml for various times (B). The survival curves were about exponential down to a surviving fraction of $10^{-3}$. Whatever the exact shape of the survival curves, both cell lines were extremely sensitive to ADM when treated *in vitro*.

### *ADM on tumours* in vivo

WHT mice with WHFIB tumours and CBA mice with CBSAF tumours at diameters of 6–8 mm were treated with ADM given i.p. at doses from 1·3 to 18 m/g kg. The $LD_{50}$ of ADM in both strains is about 18 mg/kg. Tumours were excised 2–44 h after drug injection and assayed for cell survival *in vitro*. Fig. 3 (A and B) shows that there was very little cell kill in either tumour, at any drug dose or time of excision after ADM injection.

ADM was also given i.v. at a dose of 18 mg/kg to mice bearing s.c. WHFIB tumours. Cell survival was still not reduced below 30%. Thus, failure of peritoneal absorption is not the reason for the resistance *in vivo*.

In order to test for a possible tumour-size effect on the sensitivity of tumours to ADM, mice bearing lung tumours up to a maximum diameter of 2 mm were treated with 18 mg/kg of ADM. The lungs were excised 2–24 h after the ADM was given and cell survival in the lung tumours was assayed *in vitro* (Fig. 3 (C and D). There was still very little cell kill in either tumour, and cell survival never fell below 30%.

Measurements of regrowth of s.c. CBSAF tumours after injection of ADM showed that the time taken to regrow to 2 mm above the initial diameter was 1·7

days for controls, 1·9 days for mice treated with 9 mg/kg and 2·6 days for 18 mg/kg, which were not significantly different. Analysis of the time taken to regrow to 4 mm above the treatment diameter gave 4·1 days for 18 mg/kg ADM compared with 2·9 days for controls, a delay of 1·2 days, which was just significant. For the WHFIB tumour, 10 mg/kg ADM gave no significant growth delay either at 2 mm or 4 mm. These small delays are consistent with the high level of survival seen *in vitro* after treatment *in vivo* (Fig. 3).

To test the possibility that the resistance to ADM of tumours *in vivo* was due to a resistant phase in the cell cycle, WHT mice bearing 2mm WHFIB lung nodules were treated with either 2 injections of 9 mg/kg ADM 8 h apart, or 3 injections of 6 mg/kg 8 and 6 h apart. In view of the probably long tumour half-life of the drug (Siemann & Sutherland, 1979) most cycling cells should have been exposed to the drug when they were not in a resistant phase. However, cell survival was still not reduced below 30% (data not shown).

Fig. 4.—Survival of cultured exponentially growing WHFIB cells in 0·5 μg/ml ADM, in aerobic or hypoxic conditions, two experiments; △ and ◇, hypoxic; ○ and □, aerobic.

### *Drug measurements*

Fluorometric measurements of the average amount of ADM in s.c. CBSAF tumours 4, 9 or 28 h after i.p. injection of 9 mg/kg of the drug gave levels of 12, 4 and 0·25 μg/g respectively. These levels would cause massive cell kill *in vitro*. For example, a dose of 4 μg/ml of ADM for 1 h to CBSAF cells would reduce survival well below $10^{-3}$.

### *Hypoxia*

Hypoxia has been found to be a significant factor in reducing chemosensitivity to bleomycin *in vitro* (Roizin-Towle & Hall, 1978). The WHFIB tumour has a hypoxic fraction of at least 50% at diameters of 6–8 mm, and the CBSAF a hypoxic fraction of at least 10% (McNally, unpublished). We therefore investigated the effect of hypoxia on sensitivity to ADM of WHFIB cells in suspension cul-

Fig. 5.—Survival of cultured WHFIB cells in 0·5 μg/ml ADM, in exponential phase (redrawn from Fig. 4) or plateau phase (×).

ture. Oxic and hypoxic cells were exposed to 0·5 μg/ml ADM at 37°C for times up to 4·5 h. The resulting survival curves were both biphasic (Fig. 4) with the breakpoint occurring at about 2 h. The hypoxic cells were more resistant than the oxic cells, but not enough to explain the resistance of tumour cells *in vivo.*

*Plateau phase*

When plateau-phase WHFIB cells in suspension culture were exposed to 0·5 μg/ml ADM for times up to 4·5 h, the survival curve had a $D_0$ of 6·9 h, *i.e.* the cells were very resistant (Fig. 5). The slope of the sensitive region of the survival curve for oxic exponential cells is in fact 15·3 times steeper than that for plateau-phase cells.

### DISCUSSION

The chemosensitivity of both tumour cell lines *in vitro* is of the same order of magnitude as that reported by Kim & Kim (1972) for HeLa cells, Barranco *et al.* (1973) for Chinese hamster ovary cells and Belli & Piro (1977) for V79 cells. HeLa cells, exposed to varying concentrations of ADM for 1 h, gave a linear survival curve with a $D_0$ of 0·1 μg/ml, compared to 0·11 μg/ml for the sensitive phase of the WHFIB. Chinese hamster ovary cells and V79 cells, under the same conditions, both gave biphasic curves similar to but slightly more resistant than WHFIB cells. CBSAF cells were also slightly more resistant than WHFIB cells.

The factor which we have shown most profoundly to influence chemosensitivity of cells *in vitro* to ADM is their growth phase. For plateau-phase WHFIB cells in suspension culture exposed to 0·5 μg/ml for various times, the exponential survival curve had a $D_0$ of 6·9 h (Fig. 5) compared with about 0·45 h for log-phase cells. Similar results were obtained with V79 379A cells, which could be grown in monolayer culture (Martin, unpublished).

Belli & Piro (1977) using V79 cells, and Sutherland *et al.* (1979) using EMT6 mammary tumour cells grown into spheroids, both found that plateau-phase cells were significantly more resistant to ADM than log-phase cells. Sutherland *et al.* (1979) showed by fluorescence measurements that non-dividing cells in the outer region of the spheroid took up significantly less drug than log-phase cells. They also found that, after normalizing for actual drug uptake into the cells, there was little difference between the sensitivity of exponential and plateau-phase cells. However, there were cells deeper in the spheroids, which were still more resistant, even after normalizing for drug uptakes. Their values for survival of dissociated EMT6 cells or whole spheroids exposed to 0·5 μg/ml ADM for 1 h ($10^{-3}$ and 0·3 respectively) reflected the same degrees of sensitivity in the single cells and resistance in aggregated cells that we have found in cells and whole tumours respectively.

Hypoxia is a factor known to affect profoundly the radiosensitivity of tumour cells. Were it an important factor in chemosensitivity, the high hypoxic fraction in the WHFIB tumour (50% or more) might explain the differences in chemosensitivity *in vivo* and *in vitro.* Acutely hypoxic cells *in vitro* were more resistant than oxic ones, but only to a relatively small extent (Fig. 4) and not sufficient to explain the resistance of tumours *in vivo.* Harris & Shrieve (1979) found no difference in sensitivity to ADM in either acutely or chronically hypoxic EMT6 cells when compared with oxic cells, but they exposed the cells to the drug for only up to 2 h. Our data show that it was only *after* 2 h that differences in sensitivity between oxic and hypoxic cells cells became marked. We have since found that WHFIB cells exposed to 95% $N_2$/5% $CO_2$ for up to 24 h became progressively more resistant, but still not to a degree adequate to explain resistance of tumours *in vivo* (data not shown). Smith *et al.* (1980) found that hypoxic V79 cells became much more resistant to ADM with longer times in hypoxia. However, in these circumstances one may be altering factors other than the state of oxygenation of the cells, *e.g.* nutritional status, pH, or growth fraction.

Since the sensitivity to ADM of cultured cells *in vitro* differs so much from that of the tumours *in vivo*, it is possible that the cells composing the tumours had become inherently more resistant to ADM while growing *in situ*. To test this, s.c. tumours were excised, cell suspensions prepared and appropriate numbers plated on dishes. These were then treated with ADM 16 h later. Cells from both WHFIB and CBSAF tumours gave survival curves which were similar to the corresponding survival curves for cultured cells, though in each case they were slightly more resistant. WHFIB tumour cells gave a survival curve with a $D_0$ of 0·18 μg/ml (mean of 4 experiments) compared with 0·11 μg/ml for cultured cells; CBSAF cells gave a survival curve with $D_0 = 0{\cdot}28$ μg/ml, compared with 0·14 μg/ml for cultured cells. However, this increased resistance is quite inadequate to explain the enormously greater resistance of tumours *in vivo*. If cells explanted from tumours were treated 48 h after plating, when they were in log phase, this small resistance was lost and they responded in the same way as the cultured cells. Thus, the resistance to ADM of tumours *in vivo* is probably not due to an inherent resistance of the tumour cells *in vivo*, relative to *in vitro*.

The demonstration that i.v. ADM did not increase cell kill, and that reasonably high levels of the drug were in the tumour, suggest that the lack of effect on tumours *in vivo* is not due to failure of drug delivery to the tumour as a whole. In spite of this, the drug is clearly not very cytotoxic *in vivo*.

If drug-killed cells in tumours were being rapidly removed and therefore not counted in the haemacytometer, this would inflate the estimate of cell survival in the tumour. However, in this case we should expect a decrease in cell yield in tumours from ADM-treated mice relative to control mice. Since tumour half-life is long (Siemann & Sutherland, 1979) the *in vitro* data would predict a surviving fraction below $10^{-3}$. However, survival was always greater than 30%. A fall in cell yield could only explain this if there were a loss of 2–3 decades of cells, which would certainly be detectable. In practice we have not detected any systematic fall in cell yield following treatment with ADM. The slight delay in tumour growth agrees with the high level of cell survival, and is a further indication that this was not an artefact due to loss of drug-killed cells.

The drug Amphotericin B (Fungizone) was incorporated in the medium used in these experiments at a concentration of 2·5 μg/ml. Hahn *et al.* (1977) have shown that in HA1 cells this drug caused cell kill at high temperatures, but not at 37°C. They suggested that this effect might be due to the action of the drug on the cell membrane. Therefore there is a possibility that its presence in the medium could have affected results by modifying the cell membrane and restricting entry of ADM into the cell. We feel this is unlikely for the following reasons: (1) Hahn *et al.* (1977) could demonstrate the effect only at high temperatures with higher drug concentrations than ours; (2) the difference we have seen between the response of oxic *vs* hypoxic cells and plateau versus log-phase cells occurred in cells growing in identical media; (3) log-phase WHFIB cells respond to ADM in the same way, whether or not amphotericin B is present in the medium (data not shown).

We have found that 2mm lung tumours are just as resistant to the drug as 6–8mm s.c. tumours. Thus, at least in this size range, no increase in sensitivity to ADM as tumour size decreases is seen. This has important implications in clinical chemotherapy, in which much stress is placed on the belief that micrometastases are more sensitive than the primary tumour. In breast carcinoma, ADM is commonly used in prophylactic regimes designed to treat preclinical metastases, together with other drugs, *e.g.* vincristine, methotrexate, cyclophosphamide and 5-fluorouracil. "Preclinical" lung metastases in the WHFIB tumour were clearly no more sensitive than s.c. tumours of 50–500 times the volume at the "primary" site. However, this does not preclude the possibility that in tumours which *are* sensitive to ADM there is a tumour-size effect, as there is with radiation (Shipley *et al.*, 1975) cyclophosphamide (Twentyman, 1977) and BCNU (Steel *et al.*, 1976).

It is possible that the same factors which cause the cells at the centre of a spheroid to be resistant are causing the tumours to be resistant. If the sole reason for resistance of tumours *in vivo* were that a high proportion of their cells were not cycling and so excluding the drug, we would have to conclude that even in 2mm poorly-differentiated lung tumours many of the cells were out of cycle and so resistant to the drug. Labelling studies should give more information on this. Other factors may also prevail in the inner tumour or spheroid to produce ADM resistance. These may include pH changes, prolonged hypoxia, poor nutritional status, cell contact phenomena or inhibition of drug action by the presence of breakdown products.

We should like to thank the Medical Research Council for the Fellowship which enabled this work to be carried out. We are grateful to Mrs Lynda Hall and her staff for the breeding, maintenance and care of the animals and to Mrs J. de Ronde for her expert technical assistance. We are grateful to Dr M. R. L. Stratford and Mr A. I. Minchinton for pharmacological drug assay and to Montedison Pharmaceuticals for supplying the drug. We appreciate the constructive criticism and help we received from Professor J. F. Fowler and the financial support of the Cancer Research Campaign.

### REFERENCES

BALCONI, G., BOSSI, A., DONELLI, M. G. & 4 others (1973) Chemotherapy of a spontaneous mammary carcinoma in mice: Relation between *in vitro-in vivo* activity and blood and tumour concentrations of several antitumor drugs. *Cancer Chemother. Rep., Pt 1*, **57**, 115.

BARRANCO, C., GREEN, E. W., BURK, K. H. & HUMPHREY, R. M. (1973) Survival and cell kinetic effects of Adriamycin on mammalian cells. *Cancer Res.*, **33**, 11.

BELLI, J. A. & PIRO, A. J. (1977) The interaction between radiation and Adriamycin damage in mammalian cells. *Cancer Res.*, **37**, 1624.

BENJAMIN, R. S., WIERNIK, P. H. & BACHUR, N. R. (1974) Adriamycin chemotherapy: Efficacy, safety and pharmacologic basis of an intermittent single high-dosage schedule. *Cancer*, **33**, 19.

BONADONNA, G., MONFARDINI, S., DELANA, M. & others (1972) Clinical trials with Adriamycin: Results of three years' study. In *International Symposium on Adriamycin.* Ed. Carter *et al.* New York: Springer-Verlag. p. 139.

BONADONNA, G., DELANA, M., USLENGHI, C. & others (1974) Combination therapy of advanced Hodgkin's disease (HD) with a combination of Adriamycin (ADM), Bleomycin (BLM), vinblastine (VBL) and imidazole carboxamide (DTIC) *versus* MOPP. *Proc. Am. Assoc. Cancer Res.*, **15**, 90.

CARTER, S. K. (1972) Single and combination nonhormonal chemotherapy in breast cancer. *Cancer*, **30**, 1543.

CARTER, S. K. (1975) Adriamycin—A review. *J. Natl Cancer Inst.*, **55**, 1265.

CARTER, S. K. & LIVINGSTONE, R. B. (1973) Single agent therapy for Hodgkin's disease. *Arch. Intern. Med.*, **131**, 377.

CHAN, K. K. & HARRIS, P. A. (1973) A fluorometric determination of Adriamycin and its metabolites in biological tissues. *Res. Commun. Chem. Pathol. Pharm.*, **6**, 447.

FU, K. K., BEGG, A. B., KANE, L. J. & PHILLIPS, T. L. (1979) Interaction of radiation and Adriamycin on the EMT6 tumour as a function of tumour size and assay method. *Int. J. Radiat. Oncol. Biol. Phys.*, **5**, 1249.

GEORGE, K. C., HIRST, D. G. & MCNALLY, N. J. (1977) Effect of hyperthermia on cytotoxicity of the radiosensitizer Ro 07-0582 in a solid mouse tumour. *Br. J. Cancer*, **35**, 372.

GOTTLIEB, J. A., RIVKIN, S. E., SPIGEL, S. C. & 4 others (1974*a*) Superiority of Adriamycin over oral Nitrosoureas in patients with advanced breast carcinoma. *Cancer*, **33**, 519.

GOTTLIEB, J., BODEY, G. & SINKOVICS, J. (1974*b*) An effective new 4-drug combination (CY–VA–DIC) for metastatic sarcomas. *Proc. Am. Assoc. Cancer Res.*, **15**, 162.

HAHN, G. M., LI, G. C. & SHIU, E. (1977) Interaction of Amphotericin B and 43°C hyperthermia. *Cancer Res.*, **37**, 761.

HARRIS, J. R., TIMBERLAKE, N., HENSON, P. & BELLI, J. A. (1978) Adriamycin uptake and release in V79 Chinese hamster cells. *Radiat. Res.*, **74**, 499.

HARRIS, J. W. & SHRIEVE, D. C. (1979) Effects of Adriamycin and X-rays on euoxic and hypoxic EMT6 cells *in vitro*. *Int. J. Radiat. Oncol. Biol. Phys.*, **5**, 1245.

KIM, S. H. & KIM, J. H. (1972) Lethal effect of Adriamycin on the division cycle of HeLa cells. *Cancer Res.*, **32**, 323.

ROIZIN-TOWLE, L. & HALL, E. J. (1978) Studies with Bleomycin and Misonidazole on aerated and hypoxic cells. *Br. J. Cancer*, **37**, 254.

ROSEN, G., SUNANSIRIKUL, S., KWON, C. & 4 others (1974) High-dose methotrexate with citrovorum factor rescue and Adriamycin in childhood osteogenic sarcoma. *Cancer*, **33**, 1151.

SHIPLEY, W. W., STANLEY, J. A. & STEEL, G. G. (1975) Tumour size dependency in the radiation response of the Lewis lung carcinoma. *Cancer Res.*, **35**, 2488.

SIEMANN, D. W. & SUTHERLAND, R. M. (1979) A comparison of the pharmacokinetics of multiple and single dose administration of Adriamycin. *Int. J. Radiat. Oncol. Biol. Phys.*, **5**, 127.

SMITH, E., STRATFORD, I. J. & ADAMS, G. E. (1980) Cytotoxicity of Adriamycin on anaerobic and hypoxic Chinese hamster V79 cells *in vitro*. *Br. J. Cancer*, **42**, 568.

STEEL, G. G., ADAMS, K. & STANLEY, J. (1976) Size dependence of the response of Lewis lung tumours to BCNU. *Cancer Treat. Rep.*, **60**, 1743.

SUTHERLAND, R. M., EDDY, H. A., BAREHAM, B., REICH, K. & VANANTWERP, D. (1979) Resistance to Adriamycin in multicellular spheroids. *Int. J. Radiat. Oncol. Biol. Phys.*, **5**, 1225.

SUTOW, W. W., SULLIVAN, P. & FERNBACH, D. (1974) Adjuvant chemotherapy in primary treatment of osteogenic sarcoma. *Proc. Am. Assoc. Cancer Res.*, **15**, 20.

TWENTYMAN, P. R. (1977) Sensitivity to cytotoxic agents of the EMT6 tumour *in vivo*: Tumour volume *versus in vitro* plating. I. Cyclophosphamide. *Br. J. Cancer*, **35**, 208.